# Therapeutic mammoplasty: a “wise” oncoplastic choice—lessons from the largest single-center cohort from Asia

**DOI:** 10.3389/fonc.2023.1131951

**Published:** 2023-04-14

**Authors:** Chaitanyanand Koppiker, Sneha Joshi, Rupa Mishra, Devaki A. Kelkar, Pragnya Chigurupati, Anjali Joshi, Jisha John, Shweta Kadu, Amitkumar Bagdia, Deepti Thakkar, Chetan Deshmukh, Harikiran Allampati, Gautam Sharan, Upendra Dhar, Smeeta Nare, Sanket Nagarkar, Laleh Busheri, Beenu Varghese, Mugdha Pai

**Affiliations:** ^1^ Orchids Breast Health Centre, Prashanti Cancer Care Mission, Pune, India; ^2^ Centre for Translational Cancer Research: A Joint Initiative of Indian Institute of Science Education and Research (IISER), Pune and Prashanti Cancer Care Mission (PCCM), Pune, India; ^3^ International School of Oncoplastic Surgery, Pune, India; ^4^ Jehangir Hospital, Pune, India; ^5^ Ruby Hall Clinic, Pune, India; ^6^ Department of Radiation Oncology, Inlaks and Budhrani Hospital, Pune, India

**Keywords:** therapeutic mammaplasty (TM), largest cohort from Asia, quality of life, PROMS (patient reported outcome measures), oncoplasty, breast cancer

## Abstract

**Introduction:**

The majority of breast cancer patients from India usually present with advanced disease, limiting the scope of breast conservation surgery. Therapeutic mammoplasty (TM), an oncoplastic technique that permits larger excisions, is quite promising in such a scenario and well suited to breast cancer in medium-to-large-sized breasts with ptosis and in some cases of large or multifocal/multicentric tumors. Here, we describe our TM cohort of 205 (194 malignant and 11 benign) patients from 2012 to 2019 treated at a single surgeon center in India, the largest Asian dataset for TM.

**Methods:**

All patients underwent treatment after careful discussions by a multidisciplinary tumor board and patient counseling. We report the clinicopathological profiles and surgical, oncological, cosmetic, and patient-related outcomes with different TM procedures.

**Results:**

The median age of breast cancer patients was 49 years; that of benign disease patients was 41 years. The breast cancer cohort underwent simple (n = 84), complex (n = 71), or extreme (n = 44) TM surgeries. All resection margins were analyzed through intra-operative frozen-section assessment with stringent rad-path analysis protocols. The margin positivity rate was found to be 1.4%. A majority of the cohort was observed to have pT1–pT2 tumors, and the median resection volume was 180 cc. Low post-operative complication rates and good-to-excellent cosmetic scores were observed. The median follow-up was 39 months. We observed 2.07% local and 5.7% distal recurrences, and disease-specific mortality was 3.1%. At median follow-up, the overall survival was observed to be 95.9%, and disease-free survival was found to be 92.2%. The patient-reported outcome measures (PROMs) showed good-to-excellent scores for all types of TMs across BREAST-Q domains.

**Conclusion:**

We conclude that in India, a country where women present with large and locally advanced tumors, TM safely expands the indications for breast conservation surgery. Our results show oncological and cosmetic outcomes at acceptable levels. Most importantly, PROM scores suggest improved overall wellbeing and better satisfaction with the quality of life. For patients with macromastia, this technique not only focuses on cancer but also improves self-image and reduces associated physical discomfort often overlooked by women in the Indian setting. The popularization of this procedure will enable Indian patients with breast cancer to receive the benefits of breast conservation.

## 1 Introduction

Therapeutic mammoplasty (TM), an oncoplastic technique, combines oncological safety, breast reduction, and mastopexy techniques enabling breast conservation for select breast tumors in moderate-to-large breasts. In the last three decades, breast conservation therapy (BCT), which involves breast conservation surgery (BCS) followed by radiation therapy (RT), has gained acceptance as a standard of care for breast cancer (1, 2). Several large cohort studies have shown equivalent survival rates between BCT and mastectomy with long-term follow-up (3, 4). Recent studies have also suggested better disease-free and overall survival with improvement in quality of life (QoL) in patients undergoing BCT as compared with mastectomy (5–8).

However, in cases where large excisions of the breast tissue were required, unsatisfactory cosmetic outcomes, like BCT site defects, bird-beak deformity (9), or asymmetry in breasts or nipples post-BCT, have been observed (10), thus limiting the application of conventional BCT. In addition, BCT has limited applications in patients with multifocal or multicentric (MF/MC) disease and in cases of extensive microcalcifications. Though conventionally MF/MC cancers have been labeled as a contraindication for BCS, with modern extreme oncoplasty, they can be accommodated under the TM fold (11–15).

The concept of oncoplastic breast surgery (OBS) was first introduced in the 1990s by Prof. Audretsch when he described the technique of partial reconstruction of the breast using plastic surgical (15–17). OBS is now increasingly being accepted as the standard of care in the surgical management of breast cancer cases across the world due to benefits such as oncological safety with concurrent improvement in aesthetic results and QoL (5, 8, 18).

OBS procedures involving partial breast reconstruction are classified as volume replacement or displacement techniques (9, 19). TM is a commonly used volume displacement technique suitable for OBS in women with medium-to-extra-large breasts with ptosis. TM combines the advantages of an oncologically safe wide excision of the tumor with breast reduction, mastopexy, and contralateral symmetrization techniques (20–22). In addition, TM has been shown to achieve satisfactory outcomes by reducing breast size, thereby facilitating better delivery and distribution of RT regimens, achieving contralateral breast symmetry, and improving the QoL (23). TM is reported to have recurrence rates of between 0% and 9% and shows oncological outcomes comparable to those of BCS (24–26). Furthermore, TM offers an option for BCS in women who present with locally advanced breast cancer (LABC) (Stage IIB or greater) (27) or large operable breast cancer (LOBC) (>5 cm), MF/MC, or extensive microcalcifications wherein a mastectomy would be the surgical procedure of choice (20, 22). However, even though its use has been indicated for smaller ptotic breasts in selective cases, TM may not be effective due to the paucity of breast tissue (28). Recently, data from the national iBRA-2 and TeaM studies were combined to compare the safety and short-term outcomes of TM and mastectomy with or without immediate breast reconstruction (IBR). These data indicated that BCS was possible in 87% of TM cases without delay in adjuvant treatment, indicating that TM may allow high-risk patients who are not candidates for IBR to avoid mastectomy safely (22, 29). However, the need of the hour is large, randomized trials assessing the benefit of oncoplastic techniques with long-term follow-up.

The majority of Indian breast cancer patients present with large tumors in advanced stages (30). This limits the scope of upfront BCS with or without OBS unless the patient has a favorable breast-to-tumor ratio. In such patients with large tumors but an unfavorable breast-to-tumor ratio, OBS with the TM procedure has been shown to effectively extend the boundaries of surgical excisions (31). However, the field of OBS is still nascent in India and is practiced only by a handful of breast surgeons in metropolitan cities.

With this background, we undertook the current study to investigate and analyze the outcomes of TM with a focus on oncological safety and efficacy. From our single-institutional TM cohort, we present data on 205 patients with breast disease who underwent 222 TM surgeries after analysis of the feasibility and safety of the procedure, careful counseling, and multidisciplinary team (MDT) discussion.

Based on the guidelines of the TeaM Study protocol, we report the clinicopathological profiles and oncological outcomes of our cohort and experiences related to various TM surgical techniques. In addition to being the largest single institutional study from Asia, a major asset of the study is the patient-reported outcome measures (PROMs) as well as cosmetic outcomes for a large portion of the cohort. This study also aims to provide recommendations and suggestions for breast oncosurgeons to easily adapt TM in their regular clinical practice for breast cancer management.

## 2 Methods

### 2.1 Patient selection

At our institution, detailed pre-op counseling is performed by the surgeon to discuss the various treatment and surgical options in a shared decision-making process. Patients presenting with breast disease who had moderate-to-extra-large breasts with ptosis were counseled for TM.

### 2.2 Clinical management

Triple assessment based on clinical examination, appropriate imaging, and image-guided core needle biopsy was routinely used to establish a diagnosis. Confirmed breast cancer cases underwent a breast surgery at a network hospital site. After clinical staging, patients were selected for neoadjuvant chemotherapy/neoadjuvant hormonal therapy (NACT/NAHT) and adjuvant treatment based on decisions made at MDT, in accordance with the unit’s protocol and suggested global treatment guidelines.

### 2.3 Surgical procedures

In our practice, we classify TM techniques into four categories according to the indications described in **Table 1** (9, 11, 19).

**Table 1:** Classification of TM techniques.

Type	Description	References
Simple	For tumors within the reduction pattern (i.e., at 6 o’clock) The nipple is placed on the superior, supero-medial, and inferior pedicles, which are commonly used pedicles	(Savalia and Silverstein 2016) (28)
Complex	For tumors outside the pattern of reduction (i.e., between 12–3 o’clock position (left breast) and 12–9 o’clock (right breast) Dual pedicle technique is applied in which extended and/or secondary pedicles (inferior, infero-lateral, or infero-medial) act as fillers, which enhance vascularity as an added advantage* *Extended or secondary pedicles are the other parts of the breast that are generally excised, which are used to fill the defects. The latter are preferred, as they have better blood supply reaching the most distant areas of the pedicle as compared to extended ones	(Savalia and Silverstein 2016) (28)
Extreme	Include large multicentric, multifocal tumors, extensive DCIS, and poor response to NACT requiring large areas of resection (>5 cm), in which mastectomy would be the surgical procedure of choice	(Silverstein et al., 2015, Silverstein et al., 2016, Koppiker et al., 2019) (31–33)
Split reduction	For tumors that lie outside the reduction pattern wherein the skin needs to be resected due to involvement or close proximity with tumor The lower limb of the Wise pattern is shifted over the tumor site. Then, the outer limb of the Wise pattern is shifted upward to lie over the tumor so that there is no incision in the IMF on the outer side In contrast to those of the other techniques, the incisions on the IMF and the horizontal limb on the side of the skin excision are omitted to preserve the vascularity and restructure the breast	(Silverstein et al., 2015) (12)

TM, therapeutic mammoplasty; DCIS, ductal carcinoma in situ; NACT, neoadjuvant chemotherapy; IMF, inframammary fold.

#### 2.3.1 Pre-operative markings

In the pre-operative planning, appropriate markings are made on both breasts based on a Wise pattern or vertical scar incision. The nipple–areolar complex (NAC) is re-positioned between 19 and 23 cm from the sternal notch, which is often determined by placing the fingers at the inframammary fold (IMF) and projecting on the anterior surface of the breast into the meridian.

#### 2.3.2 Tumor localization

Clinically palpable lesions are localized in the usual fashion intraoperatively. For impalpable lesions, tumor localization is performed pre-operatively by stereotactic guide-wire placement using mammography or high-resolution ultrasonography. For sono-localizable lesions, intra-op ultrasonography (USG) might be used. Post-NACT impalpable tumors may be localized with the help of marker clips placed pre/mid chemotherapy (Koppiker et al. unpublished observations).

#### 2.3.3 Incision, tumor excision, and oncological clearance

**Figure 1:**
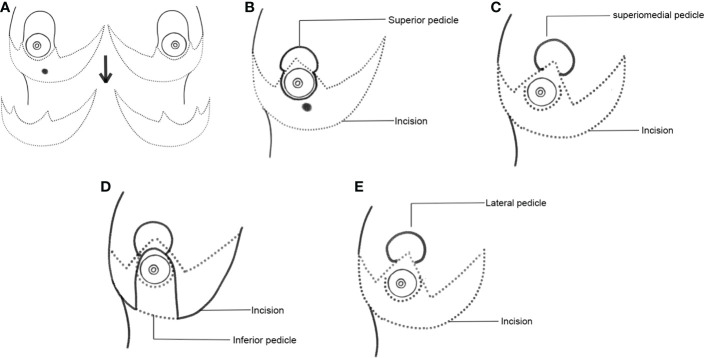
Schematic representation of Wise pattern incision and various choices of pedicles. **(A)** Wise pattern incision. **(B)** Superior pedicle. **(C)** Superior-medial pedicle. **(D)** Inferior pedicle. **(E)** Lateral pedicle.

The surgery begins by marking out the Wise pattern incision (**Figure 1A**). The Wise pattern is located to excise the localized tumor with wide margins. The area of the appropriate pedicle that will carry the nipple is marked and de-epithelized. The tumor is then excised with wide margins through one of the limbs of the Wise pattern. If required, further imaging of the specimen is performed using specimen mammography to ensure that the tumor is excised with wide margins. The shaved margins of the cavity are further excised and sent for frozen-section evaluation to ensure margin negativity and perform any cavity margin re-excision if needed. Once negative tumor margins of the excision cavity are achieved, the decision is made to use one of the appropriate pedicles.

#### 2.3.4 NAC positioning

The NAC is marked out, and an incision is carefully made around the areola. The tumor and its quadrant are then widely excised through one of the limbs of a Wise pattern incision (based on the type of TM technique decided).

#### 2.3.5 Marking out the tumor bed for targeting radiotherapy

The tumor bed is marked with Liga clips in the superior, inferior, medial, lateral, basal, and anterior margins. In our experience, the tumor margins remain contained within the initial tumor volume for targeted radiotherapy. The possibility of the tumor margin getting repositioned in some other quadrant is less likely (34, 35).

#### 2.3.6 Choice of pedicles for various TM techniques

The appropriate pedicles are marked out and dissected according to the location of the tumor. According to quadrant diagrams (**Figures 1B–E**), if the tumor is at the 12 o’clock position in the upper outer quadrant (UOQ), an extended inferior pedicle is used. If a tumor is present in the outer quadrants (i.e., at the 2, 3, or 4 o’clock position of the left breast or the 8, 9, or 10 o’clock positions of the right breast), the dual pedicle technique is preferentially used. In this technique, the inferior pedicle fills up the gap, and the NAC is positioned on a superior, superomedial, or lateral pedicle. The main aim of the dual pedicle technique is to contour the defect with one pedicle and position the NAC on the other, thereby providing a dual vascular supply.

We also discuss representative cases from each type of surgery. Simple TM typically utilizes a single pedicle and is represented in Case 1. An extended inferior pedicle or a dual pedicle provides optimal outcomes in complex TM procedures (Cases 2 and 3). Extreme or split reduction TM is a suitable option for cases with large excisions that are otherwise indicated for mastectomy (Case Study 4).

#### 2.3.7 Axillary management

Once these Wise pattern incisions are carried out through to the chest wall, the lateral dissection is taken into the axilla for axillary management through one of the limbs of the same incision (sentinel lymph node biopsy/axillary lymph node dissection (SLNB/ALND), as appropriate). No separate incision is taken on the axilla. Care is taken to dissect the lateral thoracic artery and to ensure that the lateral pillar is well-perfused by various perforators. Thereafter, the incisions are closed. Drains are not inserted in the axilla unless an axillary clearance has been performed.

### 2.4 Post-surgery protocols

#### 2.4.1 Assessment of post-surgery complications

Post-surgery outcomes were assessed by breast oncoplastic surgeons and radiation oncologists. As per the Clavien–Dindo classification, post-surgery complications were classified as “major” when they required surgical intervention and “minor” when they were managed conservatively (36). We also noted the time between the completion of surgery and the start of adjuvant therapy to ascertain any delays in adjuvant therapy.

#### 2.4.2 RT methodology

The RT dose planning was aimed at achieving a biologically effective dose (BED) of 40 Gy in 15 cycles (with an optional boost to the tumor bed, if indicated). The breast along with the supraclavicular region (if indicated) was irradiated by 6-MV photon beams using forward plan field-in-field intensity-modulated radiation therapy (F-P FiF IMRT) or volumetric modulated arc therapy (VMAT). Vac-Lok immobilization and CT-based contouring and planning were performed after target delineation after Eclipse™ treatment planning system (TPS) (Version 13.5.35) for F-P FiF IMRT plans and Monaco (Version 5.11) TPS for VMAT plans. Tangential fields with sub-fields were used for radiotherapy planning. Linac, Elekta Medical System™ (Crawley, UK) with 80-leaf multileaf collimator (MLCi) was used. RT plan was accepted if at least 95% of the prescribed dose covers 100% of the planning target volume (PTV). Hot spot in PTV was accepted up to 110% of the prescribed dose. Tumor bed boost, wherever indicated, was performed using either an electron portal or simultaneous integrated boost (SIB) technique with standard dose fractionation schedules.

#### 2.4.3 Patient-reported outcome measures

PROMs were used to evaluate patient satisfaction and QoL after TM procedures. To assess PROMs, the standardized BREAST-Q questionnaire was utilized. Higher scores indicate greater patient satisfaction and functionality (37).

### 2.5 Data collection

#### 2.5.1 Patient

Data collection was performed according to the recommendations of the TeaM study protocol. Data included demography, medical history, clinical findings, pathological reports (diagnostic biopsy and surgical histopathology including immunohistochemistry), details on neoadjuvant therapy, surgical intervention, pre- and post-operative images of patients, post-surgery complications, follow-up details, and PROMs. Clinical response (clinically complete response (cCR), clinically partial response (cPR), clinically stable disease (cSD), and clinically progressive disease (cPD)) and pathological complete response (pCR) to NACT of the primary tumor were calculated as per Response Evaluation Criteria in Solid Tumors (RECIST) criteria (V1.1) (38).

### 2.6 Survival analysis and statistics

Data were collected retrospectively from patient records. Follow-up information was taken as recorded in the patient file. The date of recurrence was taken from one of the biopsy pathologies, fine-needle aspiration cytology (FNAC), or PET reports. Overall survival was calculated as all-cause since in many cases it was difficult to ascertain if death was due to disease or other unrelated causes. The overall survival interval was taken as the time period between surgery and death. The exact date of death used in the analysis was in most cases taken as the closest approximation to the date of death as informed by relatives of the patient (especially for deaths that occurred in 2020–2021). Due to the COVID-19 pandemic, follow-up was very sparse starting from early 2020 until early 2022. This could be the cause for patients lost to follow-up since traveling was prohibited or much more challenging for a large part of this time period.

Median follow-up was calculated using the reverse Kaplan–Meir (KM) method of Schemper and Smith (39) in R. Survival analysis was performed in R Version 4.2 using the survivor and survminer packages (36, 40). Kaplan–Meir plots were plotted using ggpubr. Percent disease-free and percent overall survival were derived from the survival table when the time was the closest median follow-up.

## 3 Results

### 3.1 Overview of TM study cohort: characteristics of study cohort

**Figure 2:**
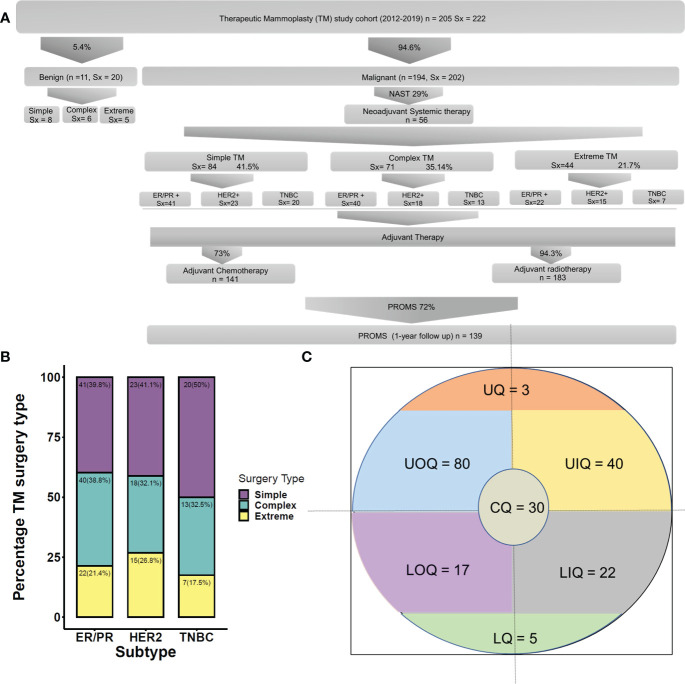
**(A)** Clinicopathological features of the cohort. **(B)** Quadrant-wise tumor location. CQ, central quadrant; LQ, lower quadrant; LIQ, lower inner quadrant; LOQ, lower outer quadrant; UIQ, upper inner quadrant; UOQ, upper outer quadrant; UQ, upper quadrant. **(C)** Molecular subtype-wise distribution of various TM techniques. TM, therapeutic mammoplasty.

The demographic distribution of study participants and their clinicopathological characteristics are summarized in **Figure 2A** and **Tables 2**–**5**. At our center, a total of 222 TM procedures were performed on 205 patients with moderate and large breasts with various grades of ptosis during 2012–2019. Among the 205 patients, 178 were unilateral breast cancer patients, 10 patients were identified with unilateral benign disease and 17 had bilateral breast disease. Among the 17 bilateral cases, eight were bilateral breast cancer cases, eight patients presented with one side benign and one side malignant, and one patient presented with bilateral benign disease. The median age at diagnosis of patients with breast cancer was 49 (29–75) years, while patients with benign breast disease had a median age of 41 (28–60) years at diagnosis. As observed in previous reports (40), a proportion of the breast cancer patients (i.e., 77/194, 40%) had comorbidities such as diabetes, making them poor candidates for a mastectomy with immediate reconstruction.

**Table 2:** Demographic distribution of breast cancer patients.

Feature	Class	N (194)
Age (years)	Median (range)	49 (29, 75)
	<40	45
	41–60	117
	>60	31
Comorbidities	Yes	77
	No	115
	NA	2
Size of breast	S	0
	M	75
	L	104
	XL	9
	NA	6
Ptosis	I	19
	II	69
	III	98
	No	3
	NA	5
Molecular subtype	ER/PR	99
	HER2	55
	TNBC	40
NACT response		N = 56
Clinical	cCR	6
Clinical	cPR	37
Clinical	cSD	3
Clinical	cPD	4
	NA	6
Pathological	pCR	16

ER, estrogen receptor; PR, progesterone receptor; TNBC, triple-negative breast cancer; NACT, neoadjuvant chemotherapy; cCR, clinically complete response; cPR, clinically partial response; cSD, clinically stable disease; cPD, clinically progressive disease; pCR, pathological complete response. NA, Not Available.

**Table 3:** Clinical features of surgeries for breast cancer.

Feature	Class	Surgeries (202)
Clinical tumor size (cT)	cT1	68
	cT2	111
	cT3	12
	NA	11
Tumor grade	I	12
	II	117
	III	56
	NA	17
Type of tumor (biopsy)	IDC	164
	IDC + DCIS	22
	ILC	3
	ILC + LCIS	3
	DCIS	8
	NA	2
Focality	Unifocal	165
	Multifocal/multicentric (MC/MF)	34
	NA	3
Quadrant	CQ	30
	LIQ	22
	LOQ	17
	LQ	5
	UIQ	40
	UOQ	80
	UQ	3
	NA	5
Pathological tumor stage	0	23
	IA	33
	IB	6
	IIA	77
	IIB	31
	IIIA	17
	IIIB	2
	IIIC	11
	IV	2

IDC, invasive ductal carcinoma; DCIS, ductal carcinoma in situ; ILC, invasive lobular carcinoma; LCIS, lobular carcinoma in situ; CQ, central quadrant; LIQ, lower inner quadrant; LOQ, lower outer quadrant; LQ, lower quadrant; UIQ, upper inner quadrant; UOQ, upper outer quadrant; UQ, upper quadrant. NA, Not Available.

**Table 4:** Demographic features of patients with benign breast disease.

Benign cases		
Feature	Class	N
Cases	Total	11
Age (years)	Median (range)	41 (28, 60)
	<40	4
	40–60	7
	>60	0
Comorbidities	Yes	1
	No	10
Size of breast	S	0
	M	3
	L	7
	XL	0
	NA	1
Ptosis	I	0
	II	2
	III	8
	No	0
	NA	1

NA, Not Available.

**Table 5:** Clinical features of surgeries for benign disease.

Benign surgeries		
Feature	Class	Surgeries (Sx = 20)
Type of tumor (biopsy)	Benign phyllodes	5
	Benign intraductal papilloma	3
	Fibroadenoma	9
	NA	3
Focality	Unifocal	15
	Multifocal/multicentric (MC/MF)	4
	NA	1
Quadrant	CQ	4
	IQ	1
	LIQ	0
	LOQ	0
	LQ	1
	UIQ	2
	UOQ	8
	UQ	1
	NA	3

CQ, central quadrant; IQ, inner quadrant; LIQ, lower inner quadrant; LOQ, lower outer quadrant; LQ, lower quadrant; UIQ, upper inner quadrant; UOQ, upper outer quadrant; UQ, upper quadrant; NA, Not Available.

Among 194 breast cancer patients (quadrant-wise tumor location is represented in **Figure 2B**), 64.4% of tumors were observed in the upper quadrant. Of 222 TM procedures (breast cancer and benign cohort together), simple TM accounted for 92 (eight benign) surgeries, while 77 (six benign) complex and 49 (five benign) extreme surgeries were performed. Subtype distribution for the different surgeries among breast cancer patients is shown in **Figure 2C**. The median pathological tumor size was 25 mm (range 2–85 mm), and the median resection volume was 180 cc. Of our 194 breast cancer patients, 56 patients received NACT, 141 ACT, and 183 RT.

### 3.2 Neoadjuvant chemotherapy (NACT/NAHT)

Among the 56 patients who received NACT, pCR was observed in 28.6% (16/56) patients. The distribution of response to NACT is given in **Table 2**. Our extreme oncoplasty cohort comprised 44 breast cancer patients of whom 18 received NACT and 4/18 showed pCR to NACT.

### 3.3 Surgical outcomes

#### 3.3.1 Surgical margins and nodal clearance

The Wise pattern technique was used in 90.1% (181/202) of therapeutic procedures for breast cancer. Clear margins were achieved in all the cancer patients with only three of 194 (1.4%) cases having positive margins. Re-excision of margins was carried out in one patient, one patient underwent an immediate complete mastectomy, and one received an additional boost to the tumor bed. Sentinel node biopsy was performed in 121 (60.2%) and axillary lymph node dissection in 83 (41.3%) of the 202 malignant surgeries.

#### 3.3.2 Post-operative complications

**Figure 3:**
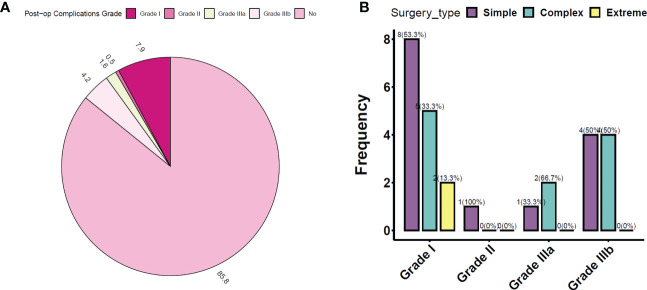
**(A)** Post-op complications observed in the study cohort (in percentage). **(B)** Distribution of post-op complications according to TM surgery type. TM, therapeutic mammoplasty.

Post-operative complications were classified based on grades as per the Clavien–Dindo classification adapted for breast cancer (36). A total of 27/194 (14%) cases of complications were observed, similar to observations reported in earlier literature (2) (**Figures 3A, B** and **Table 6**). All complications were treated conservatively in the outpatient setting. In general, we observe immediately post-surgery a low rate of Grade I/II complications even with complex and extreme mammoplasty techniques.

**Table 6:** Post-op complications in the cohort as per the Clavien–Dindo classification.

Characteristics	Complications, number (n = 194) No complications in n = 165, NA=2			
Grades	Total (27)	Simple TM (13)	Complex TM (11)	Extreme oncoplasty (2)
**Grade I (**seroma/hematoma not requiring drainage, minor skin necrosis, fat necrosis, delayed wound healing)	15	8	5	2
**Grade II** (wound infection)	1	1	0	0
**Grade IIIa (**seroma/hematoma were drained under USG guidance, lymphedema, nipple necrosis, skin necrosis undergoing debridement)	3	1	2	0
**Grade IIIb** (seroma/hematoma drained under general anesthesia—major skin necrosis, wound infection requiring debridement, bleeding)	8	4	4	0
**Total**	27	14	11	2

TM, therapeutic mammoplasty; USG, ultrasonography.

### 3.4 Adjuvant radiotherapy

Of the 194 breast cancer patients included in our study cohort, 183 patients underwent RT as clinically indicated. Among those who did not receive RT, 11 patients did not comply with the RT treatment protocol. Among those who received RT, 46 did not have any adverse reactions to the RT, 113 developed Grade I–II reactions, while only five patients developed Grade III reactions. For 19 patients, post-RT complications were not reported in our data sources. The RT regimen for various types of TM procedures was thus considered effectively safe.

### 3.5 Survival outcomes

**Figure 4:**
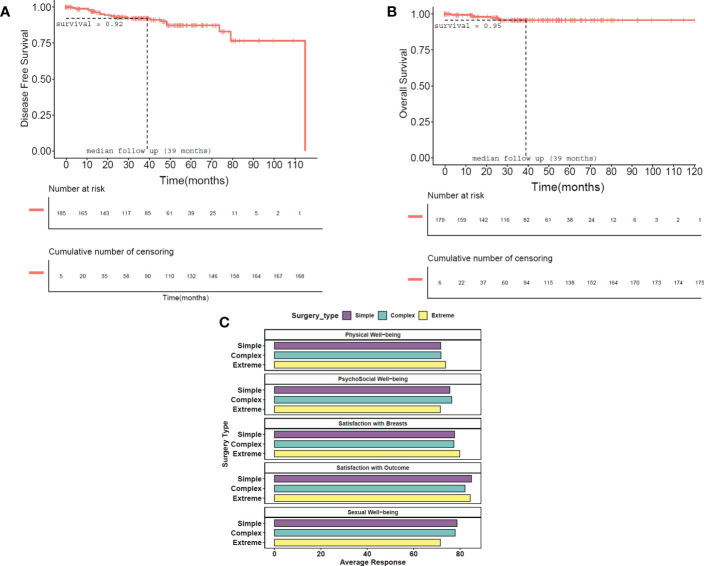
Survival Kaplan–Meir plots of disease-free and overall survival. **(A)** In disease-free survival, local and distant recurrences and metastasis are taken as events. Time is the time period in months between surgery and the known date of evidence of recurrence and is shown in months. The probability of a patient being disease-free at median follow-up (39 months) is 92.2%. **(B)** In overall survival, the approximate date of death due to any cause was taken as an event. Time is the time period in months between surgery and the approximate cause of death and is shown in months. The probability of survival at median follow-up (39 months) is 95.9%. Vertical bars indicate censored patients in both plots. **(C)** PROM scores according to TM surgery type represented graphically. Overall PROM scores are presented in the table. PROM, patient-reported outcome measure; TM, therapeutic mammoplasty.

The median follow-up was 39 months. We observed four local (2.07%) and 11 distant recurrences (5.7%), with overall recurrence at 7%, over the complete follow-up available at the time of this report. Overall mortality was 3.6% (7/194), while disease-specific mortality was only 3.1% (6/194). At the median follow-up, the overall survival probability was found to be 95.9%, with all reported deaths occurring before the median follow-up. In addition, the disease-free survival probability at median follow-up was 92.2%. KM plots of overall survival and disease-free survival are shown in **Figures 4A, B**.

### 3.6 Cosmetic score analysis

Out of 202 TM surgeries for 194 breast cancer patients, cosmetic scores were assessed by surgeons within 3–6 months post-surgery. **Table 7** shows the cosmetic scores as reported by the surgeons. Satisfaction with breasts in the PROM analysis showed an average score of 78%.

**Table 7:** Cosmetic scores for surgeries in breast cancer patients.

Category	Score	Classification	Number, (%)
1	0–3	Bad	0, (0)
2	4–5	Fair	6, (3.5%)
3	6–8	Good	117, (68.4%)
4	9–10	Excellent	48, (28.1%)
		Total	171
5	NA	Not available	23

NA, Not Available.

### 3.7 Patient-reported outcome measures

PROM data were collected from the study participants after a minimum period of 12 months post-surgery using the BREAST-Q questionnaires. Out of 194 breast cancer patients, 139 (72.0%) responded to the questionnaire. High patient satisfaction scores were observed from our PROM data as seen in **Figure 4C**.

## 4 Discussion

The TeaM publication established a comprehensive protocol for extending indications of breast conservation through mammoplasty techniques for breast cancer patients who needed wider excisions. However, there were a few limitations to the report, as it was an analysis of short-term outcomes of the practice (41, 42). Here, we present the first comprehensive, globally largest single-institutional study of 222 TM surgeries in 205 patients from 2012 to 2019 with breast disease based on the recommendations of the TeaM protocol. The major strengths of our study include the largest cohort from a single surgeon unit from a middle-income country, assessment of oncological outcomes, and cosmetic outcomes along with patient-reported outcomes.

TM is a well-established oncoplastic technique that combines the advantages of an oncologically safe wide excision of tumors with breast reduction, mastopexy, and contralateral symmetrization techniques. It extends the indications of breast conservation by enabling wider excision margins, lower re-excision rates, and a reduction in the rate of mastectomies (25). TM has been shown to achieve satisfactory outcomes by reducing the breast size, thereby facilitating better delivery and distribution of RT regimens, achieving contralateral breast symmetry, and improving the QoL. The oncological safety and efficacy of TM have been confirmed in early breast cancer cases indicated by higher rates of overall survival (OS) and disease-free survival (DFS) with low recurrence, lower complication rates, and superior cosmeses (21, 25, 43). In the Indian context, wherein mastectomy is still the default approach to breast cancer management, it is essential to incorporate oncoplastic techniques like TM in the surgical management protocol and offers more options for breast conservation.

### 4.1 Oncoplastic breast surgery and its relevance in Indian scenario

With many studies showing equivalent oncological outcomes for BCT compared to mastectomy, globally, BCT and oncoplasty have become a regular practice in the surgical management of breast cancer (41, 42). However, India brings about its own challenges, as the clinical, psychosocial, and economic profiles of breast cancer patients in India are significantly different than in Western countries. It is imperative that India rises above its current rigid mindset of mastectomy as the primary approach to breast cancer management. Another major contributor to this rigid mindset is the fact that India is a low- and middle-income country (LMIC), and for most of its population, any disease brings fear of an economic burden, aggravated by other factors like lack of education and low socio-economic status (44). Most Indian women not only are unaware of available healthcare options but also lack information regarding disease symptoms, screening modalities, self-breast examination, and/or routine mammographic screening due to societal circumstances and conservative social structure. This ultimately results in costly delays in diagnosis and treatment (45). Oncoplastic procedures are considered to be relatively expensive, and many women are not able to afford these procedures. As a result, total mastectomy is the standard of care, the rate of BCS is low, and mastectomy remains the most common option in many Tier II and III cities. BCT or OBS options are offered only by a few reputed tertiary cancer care centers in Tier I cities. The low rates of BCS are further augmented by the paucity of skilled oncoplastic surgeons in smaller centers in addition to a lack of awareness about OBS in the medical fraternity. Hence, it is essential that more surgeons are given an opportunity to train in oncoplastic techniques (45, 46).

Thus, the need for standard OBS procedures modified to fulfill the requirements of Indian breast cancer patients is even more pertinent. Taking into consideration multiple factors relevant to the Indian population, we have developed specific patient-related decision-making algorithms. These algorithms include extensive MDT discussions with a focus on the tumor location and breast size as well as patient counseling. We have developed a meticulous counseling protocol that concentrates on the psychology of the Indian woman. The counseling involves discussion of the associated pros and cons of available surgical options that enable patients to make an informed decision regarding their treatment.

Surgical management of breast cancer the world over has shifted focus from mere survival to post-breast cancer patient quality of life. Our oncoplastic practice believes that Indian breast cancer patients should also be given the opportunity to avail the benefits of breast conservation and when needed oncoplastic techniques. This will enable them to not only lead an oncologically safe life but also be cosmetically at-par. In pre-surgery counseling, our patients are made aware of the various options available to them and the advantages and disadvantages of each. Our patients are also counseled about the fact that it is necessary to think about life beyond the disease and consider the repercussions of mastectomy on their quality of life.

### 4.2 TM algorithm at our single surgeon center

Our study represents the first detailed report on surgical, oncological, and PROM outcomes after TM surgery in breast cancer patients from India as observed in a single breast oncoplasty unit.

MDT-based decisions and patient counseling identified TM as the most appropriate surgical approach for the 205 patients (194 malignant and 11 benign) in our cohort. Among these, 56 (29.01%) received NACT. In our post-NACT subset, 28.6% (16/56) had a pCR to NACT. For patients who had large residual tumors (>4 cm) post-NACT (6/56, 10.7%) an oncoplastic technique like TM, where large excisions can be achieved, facilitated breast conservation. Interestingly, in our NACT subset, a large majority of NACT non-responders were also able to undergo breast conservation through extreme oncoplastic procedures. In a parallel study from our center focused on post-NACT surgical protocols, TM was found to be an essential oncoplastic tool for successful breast conservation (Koppiker et al., manuscript in preparation).

TM gives superior cosmetic outcomes for ptotic breasts (Grades I–III) and moderate-to-large-sized breasts (47, 48). However, owing to financial and logistical challenges, Indian patients have reduced acceptance of a second operative procedure (49). We hence perform a single-step TM procedure that involves simultaneous reconstruction of the NAC and contralateral reduction mammoplasty for bilateral symmetrization in which the nipple may undergo resection with a NAC graft. Most of the cases in our study cohort have been operated using the dual pedicle technique in which NAC was carried out on the superior pedicle and the inferior pedicle was used to fill the defect caused by the excision of the tumor. In patients with smaller breasts or with large excisions, we have frequently used the whole lower segments of the breast (i.e., infero-medial and infero-lateral pedicles), so the breast mound is advanced into the defect and NAC is reimplanted onto the pedicle. TM also improves self-image and reduces physical discomfort, especially for women with macromastia, which is often overlooked by women in the Indian setting. If the patient does not consent to opposite symmetrization, alternative OBS procedures to TM are recommended.

TM has a potential advantage in achieving lower rates of re-excision due to the scope of excising wider margins (50). However, re-excision in the case of TM could also be challenging due to glandular re-arrangement during mammoplasty. This should be considered carefully after discussing within the MDT and only if the operating surgeon is confident in identifying the tumor bed and orientation (51). The TeaM protocol showed a 21% margin positivity rate, while literature reports indicate rates of positive margins ranging from 0% to 36% (22, 52) with institutions reporting lower rates of margin positivity, after conducting intra-operative frozen-section analysis (53–55). Consistent with previous reports (32), with the inclusion of intra-operative frozen-section analysis, we were able to achieve lower margin positivity with 1.4% of cases having positive margins.

Consensus guidelines for optimal RT planning after oncoplastic procedures are unclear, and further methodical investigations are needed. Indeed, results are eagerly awaited from the MIAMI trial, which is the first randomized trial designed to address the clinical safety of TM associated with the excision of each cancer and the possibility of performing up to two tumor bed(s) boost(s) radiotherapy (56).

In our study, the mean duration from TM to the start of adjuvant treatment was 50 days without any delay. This observation is consistent with several studies that indicate OBS does not result in a delay in adjuvant treatment. Although the optimal duration between OBS and RT has not yet been established, in our practice, we prefer commencing RT within 6 months of treatment during which adjuvant chemotherapy is administered, wherever recommended. If no ACT is required, RT is started within 5–6 weeks post-op.

### 4.3 Therapeutic mammoplasty in extreme situations

In the recent past, several studies have now emerged where authors have reported acceptable oncological results with equivalent survival combined with much improved cosmetic results and QoL with OBS (48, 57, 58). This has finally culminated in the concept of extreme oncoplasty (EO) where large, multicentric, and multifocal tumors as well as extensive ductal carcinoma *in situ* (DCIS) have been effectively treated with BCS.

Prof. Melvin Silverstein first introduced the concept of extended resections using oncoplastic surgery and introduced the term *extreme oncoplasty* (11). Extreme oncoplasty patients are generally those with large tumors (>5 cm), MC/MF disease, locally advanced breast cancer, or recurrences in previously irradiated breasts.

In the Indian scenario, extreme oncoplasty holds special relevance. Several reports indicate that the majority of breast cancer patients in India are diagnosed at an advanced stage, with large-sized LABC or LOBC (59). For patients with large tumors and MC/MF disease, the surgical choice of EO looks very promising. The EO technique allows the resection of larger amounts of breast tissue with safer margins and acceptable aesthetic results, thereby increasing breast conservation rates (33, 60, 61).

Our cohort includes 48 (five benign) patients who underwent extreme oncoplasty of whom 30 (62.5%) underwent an upfront extreme procedure and 18 (37.5%) received NACT followed by extreme TM. Based on our experiences, we propose that EO surgery has excellent applications for OBS-based clinical management.

### 4.4 Post-surgery commentary: oncological and cosmetic outcomes

Many studies report complication rates between 15% and 30% for OBS (62, 63). Comparable to published literature from Western cohorts, we observed lower rates of complication (13%) with a majority being only minor complications. Similarly, recurrence rates in OBS have been reported to range from 0.5% to 12%, and we observed lower rates of recurrence with only 2% local recurrence and 7% overall recurrence in our cohort. In keeping with the literature, we report 3.6% overall mortality and only 3.1% disease-specific mortality (64, 65). Although TM is an established technique and is widely practiced as a standard of care in developed nations even in high-risk patients (40, 66), it is still finding its ground in developing countries. Our encouraging results with equivalent oncological outcomes suggest the adaptability of TM as an oncoplastic technique even in low-resource settings.

Cosmetic assessment by surgeons indicated that over 80% of cases exhibited good-to-excellent cosmetic outcomes. This cosmetic assessment is mirrored in patients’ perspective as well, as we report high levels of satisfaction, with over 83% mean score of patient satisfaction with outcomes on BREAST-Q PROMs, which is expected (67), as the aim of TM is to provide an aesthetically pleasing breast along with oncological safety. In our study, a comparison of the PROMs among the types of TMs demonstrates almost equal scores, indicating that all types of TMs were well accepted. Our analysis also reveals a higher mean score of 77.05% for sexual wellbeing, which may be attributed to better body image and self-esteem arising from the satisfactory outcomes of the TM procedure and contralateral reduction mammoplasty. This is in line with previous reports that have indicated that satisfaction with breasts was better in women who underwent OBS than in those who underwent a BCT alone (29).

### 4.5 Single institution, single surgeon practice—advantages

One of the many advantages of our institution is that it is a single surgeon practice and hence brings with it benefits such as improved level of patient engagement and involvement in shared decision-making, streamlined standard operating protocols, dedicated tumor board, reduced treatment delays, and a better understanding of the patient pathway. In fact, many benefits have been associated with the independent practice that contributes to more satisfied providers, successful practice management, and higher quality care for patients. Our encouraging results could thus be credited to a multitude of factors at our institution such as quality counseling, shared decision-making, surgical expertise, a dedicated medical and surgical team, and even nursing staff that has gained experience and expertise over years. This cohesive and proficient setup contributes to the lower complication rates, personalized hospital services, and comprehensive post-operative care provided at our center.

Protocols and surgical techniques established here along with PROMs would be a useful framework for replication by other breast units. As discussed, there is a paucity of trained oncoplastic surgeons and therefore an inherent need for a structured oncoplastic training program in the country. With this aim, to fill existing gaps in breast cancer surgical training in the niche field of oncoplastic breast surgery, a sister organization of our institution, the International School of Oncoplastic Surgery (ISOS), was founded in 2014, and a structured Masters in Oncoplastic Breast Surgery program in association with University of East Anglia (UEA), UK, was initiated. The program allows budding young surgeons to gain hands-on experience and training in oncoplastic techniques specific to the Indian scenario.

If the techniques and outcomes of OBS are popularized and the broad indications of TM are clearly defined, it is possible that more eligible breast cancer patients will receive the benefits of this procedure over the routine practice of mastectomy.

### 4.6 Surgeon’s recommendations

- Careful marking placement so that the closures are not tight.
- The tumor excision should be maximum through one limb of the incision, and the axilla should be accessed through the same incision by identifying the lateral border of the pectoralis major and minor.
- The supero-lateral area and the lateral pillar should be carefully mobilized to prevent devascularization from the lower lateral segment.
- SLNB should be performed through the same incision using indocyanine green (ICG) or nuclear dye, and if the status is positive, axillary dissection should be carried out *via* the same incision.
- All the tumor margins should be analyzed on frozen sections and by a specimen mammogram. The breast restoration should be delayed until the results on frozen sections are negative. The contralateral reduction should be performed while frozen-section analysis is ongoing.
- The interruptive sutures should be used at the “T” junction instead of continuous sutures to minimize necrosis.

### 4.7 Post-surgery radiation therapy recommendations

In our opinion, in the majority of TM cases, margins around the tumor bed do not shift significantly due to the following reasons:

- During TM, adequate care is taken to check whether the tumor bed is well delineated with markings by Liga clips, as soon as the tumor is removed.
- In some cases, the margins may get advanced into the tumor cavity to form the bed of the tumor cavity (such as in an extended inferior pedicle). Herein, for dealing with the tumor in the superior quadrant, the lower margin (which is the highest point of the extended pedicle) shifts into the tumor cavity, where exactly the boost is required.
- In simple mammoplasty (or tumor in the lower quadrant or superior quadrant), in which the tumor is in a tissue segment within the specimen, it is likely that some of the margins may shift into the tumor cavity but not shift away from it.
- If the tumor is lying outside (i.e., in the outer quadrant or supero-medial quadrant) and if the excision is large, central mound advancement can be performed to fill up these cavities. In this situation, even if the infero-medial margin may shift, being a supero-medial margin, it will not go outside the tumor cavity.
- For the cavity on the outer side, if a dual pedicle technique is applied, even then inferior pedicle will be used only to fill in the gap.

## 5 Conclusion

Therapeutic mammoplasty is a promising and safe approach to manage breast cancer in medium-to-large breasts with ptosis even in the Indian context despite the scope and limitations. However, sociodemographic factors like its availability, feasibility, and resource constraints severely limit its uptake by providers and utilization by patients. Despite this, TM holds a potential promise of delivering the goal of good oncological outcomes with aesthetically pleasing results without detrimentally affecting the course of adjuvant therapies.

Our study shows promising results for the adoption of TM in routine surgical practice in India. However, given the large variability in sociocultural, psychological, and economic ground realities of the general Indian population, similar TM-focused studies from Indian breast units as well as other parts of Asia are needed to corroborate the observations from our study.

We conclude that our TM technique(s) may be suited even for advanced-stage patients with moderate-to-large breasts with mild/severe ptosis. In general, our study observations are compliant with the guidelines of TeaM protocol except for a few non-compliances such as the lack of MRI, which has poor uptake in India due to cost barriers. At our center, we were able to mitigate this challenge by doing a detailed assessment using USG and 3D tomosynthesis. Additionally, we emphasize the need to include cosmetic and PROM outcomes to assess the efficacy of TM as a viable surgical option for breast disease patients from India.

## 6 Case discussion

### 6.1 Case I: Simple Therapeutic Mammoplasty

A patient aged <35 years (Grade II ptosis) presented with a lump in the left central quadrant. USG revealed a unifocal tumor that extended from the 11 o’clock position to the 12 o’clock position, taller than wider in shape, measuring 21 × 17 × 19 mm. Tru-Cut biopsy report revealed invasive ductal carcinoma (IDC) with triple-negative breast cancer (TNBC): estrogen receptor (ER) negative, progesterone receptor (PR) negative, and HER2 negative.

Pre-operative marking was performed using a Wise pattern incision with a plan of an inferior pedicle mammoplasty. The tumor was widely excised *via* the limbs of the marked incision with the volume of excision 8 × 7 × 2.5 cm (85 g). Margin negativity was confirmed radiologically using a specimen mammogram, and the shave margins analyzed on the frozen section were negative. The tumor bed was clipped with Liga clips. The sentinel node was dissected through the same incision and was node negative. An extended inferior pedicle was mobilized to fill the defect. Thereafter, the axilla was closed, and the inferior pedicle was fixed to the chest wall. The two pillars were brought together, and the nipple–areola was sutured. The left breast tissue was reshaped and reconstructed. Contralateral reduction mammoplasty was performed on the opposite breast (right side). The post-op histopathology revealed Grade III IDC with foci of DCIS of solid and comedo type with high nuclear grade and a lesion spanning 25 × 20 mm in the central quadrant. The patient received adjuvant chemotherapy (AC-4q + paclitaxel-12q) followed by adjuvant RT with a simultaneous electron boost to the tumor bed. The patient tolerated treatment well. Genetic testing had revealed BRCA2 pathogenic mutation in the patient, and she was thus advised and underwent a prophylactic salpingo-oophorectomy. Given the high-risk status of the patient, the PCCM team has ensured diligent follow-up with routine mammograms and check-ups for the patient. She is doing well and has not developed any abnormalities or recurrence at the latest follow-up 5 years post-diagnosis.

**Figure 5:**
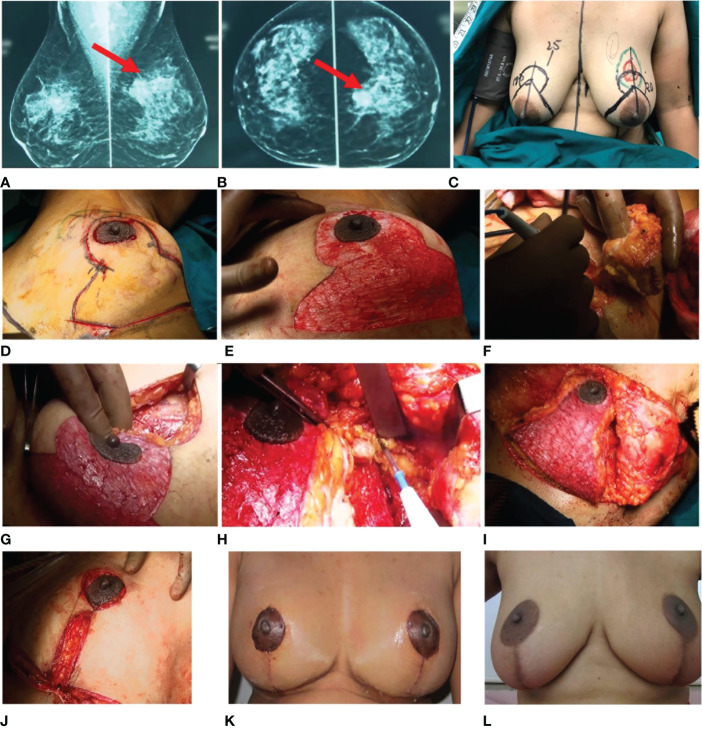
Simple TM. Pre-operative radiology images **(A, B)** show a partially circumscribed obscured iso- to hyperdense lesion seen in the upper central quadrant of the left breast. **(C)** Markings. **(D–J)** Intra-operative images. **(D)** Wise pattern incision marked. **(E)** De-epithelialized area. **(F)** Excision of the tumor. **(G)** Tumor bed. **(H)** Sentinel node biopsy. **(I, J)** Inferior pedicle is used to restructure the breast. **(K, L)** Post-operative images. **(K)** One-month follow-up. **(L)** Annual follow-up. TM, therapeutic mammoplasty.

The case images for this patient are depicted in **Figure 5**, and the technique is demonstrated in **Supplementary Video 1**.

### 6.2 Case II: Complex Therapeutic Mammoplasty

A patient aged above 60 years, with Grade III ptosis, presented with a large diffuse lump in the right UOQ. Breast radiology revealed a hypoechoic lesion measuring 28.2 mm × 16.2 mm at 12.5 o’clock 2B position along with suspected right axillary lymphadenopathy. Tru-Cut biopsy suggested Grade II invasive lobular carcinoma (ILC), and immunohistochemistry (IHC) reports indicated ER/PR-positive, HER2-negative status.

The patient was marked for a Wise pattern incision followed by excision of the large area in the UOQ. The volume excised was 8.5 × 10.5 × 5.5 cm. Specimen mammography was performed to confirm the complete removal of the tumor. The tumor bed was clipped with Liga clips. The shave margins on the frozen-section evaluation were reported as negative. The marked area for the inferior pedicle including the medial and lateral wings was de-epithelialized. The tumor was excised *via* the marked incision, and the base was clipped. The skin over the lower, medial, lateral, and superomedial quadrants was mobilized in the mastectomy plane. An extended inferior pedicle was used to fill the defect in the UOQ. Further axillary dissections were performed through the same incision. Even though the tumor location was close to the nipple, the nipple core and margins of the NAC were negative for DCIS on frozen sections. Contrary to common practice, we marked the future nipple–areola complex after mobilization and restructuring to avoid any deviation of the nipple. The right breast was reshaped and closed in two layers. A contralateral symmetrization procedure was performed.

**Figure 6:**
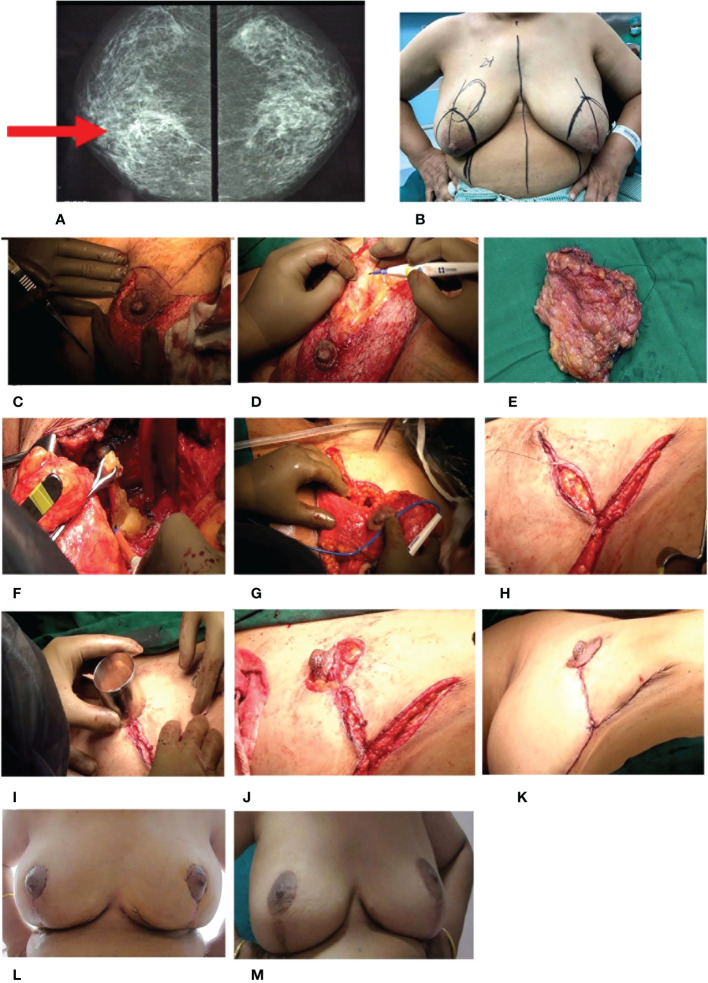
Complex TM. **(A)** Pre-operative radiology image: craniocaudal view of both breasts. Ill-defined radiodensity with spiculations and surrounding architectural distortion is seen in the deep central quadrant of the right breast. Pleomorphic amorphous microcalcification is also seen. **(B)** Markings for Wise pattern incision of the right with the tumor and contralateral breast for symmetrization. **(C–K)** Intra-operative images. **(C)** De-epithelialization of the inferior pedicle. **(D)** Excision of the tumor. **(E)** Excised tumor specimen. **(F)** Sentinel node biopsy. **(G)** Extended inferior pedicle to fill the defect. **(H)** Re-structuring of the breast. **(I)** Marking of the future nipple–areolar complex (NAC). **(J, K)** Re-structured breast with the final outcome. **(L, M)** Post-operative images. **(L)** One-month follow-up. **(M)** Annual follow-up. TM, therapeutic mammoplasty.

The post-op histopathology revealed Grade II IDC. The patient received adjuvant RT. The patient was counseled for adjuvant therapy and chose to have adjuvant endocrine therapy. The patient tolerated treatment well and is disease-free after 4 years post-diagnosis. The pre- and post-operative images for this patient are depicted in **Figure 6**, with the technique demonstrated in **Supplementary Video 2**.

### 6.3 Case III: Extreme Therapeutic Mammoplasty

A patient aged approximately 40 years with Grade II ptosis presented with a large diffuse lump in the right lower outer quadrant (LOQ). Mammogram revealed an MF (multifocal) tumor occupying a large area of the outer quadrant at the 8 o’clock position measuring 17 × 12 mm with multiple enlarged axillary lymph nodes. USG-guided core biopsy suggested IDC Grade II and IHC revealed ER/PR-positive status. HER2-positive status was confirmed by fluorescence *in situ* hybridization (FISH). The patient underwent lumpectomy twice with axillary node dissection at an external surgery site. The histopathology report showed IDC Grade III + extensive DCIS with positive margins. Axillary lymph nodes 27/32 were positive.

Before she was referred to our clinic, she underwent external site surgery, with a wide local excision of Ca breast (right). The histopathology report revealed that margins were negative for the tumor except for the lateral margin, which was positive. The patient received adjuvant chemotherapy (paclitaxel + Herceptin 12 cycles followed by AC regimen for four cycles followed by completion of Herceptin regimen). The patient was advised for mastectomy at the external site.

After referral to our hospital, the ultrasound showed a large cavity with microcalcifications extending to the lower and mid-outer quadrants in the right breast and suspicious microcalcifications in the lower quadrant of the left breast. A stereotactic vacuum-assisted biopsy was performed on the left breast, and the histopathology report revealed no malignant disease. Clips were placed at the site of the biopsy. Thereafter, a right extreme TM was performed at our hospital. The patient was marked for a Wise pattern incision, and a wide excision of the outer quadrant was performed to excise the whole cavity along with calcifications with good and adequate margins. The specimen excised was 4 × 3 × 0.5 cm, 8 × 8 × 0.5 cm, 15 × 10 × 6 cm (900 cc). Intraoperative radiology was performed to ascertain the complete removal of microcalcifications. Shave margins were sent for frozen-section evaluation and were reported to be negative. The NAC was carried out on the supero-medial pedicle, and the inferior pedicle was used as a filler to restructure the breast. Since the woman had Grade II ptosis, the length of the inferior pedicle was adequate to reach the area of excision. The right breast was restructured and closed in two layers.

On the left, the remaining microcalcification was excised under wire guidance to reconfirm the diagnosis on the frozen section. As it was benign, a contralateral symmetrization procedure was performed.

**Figure 7:**
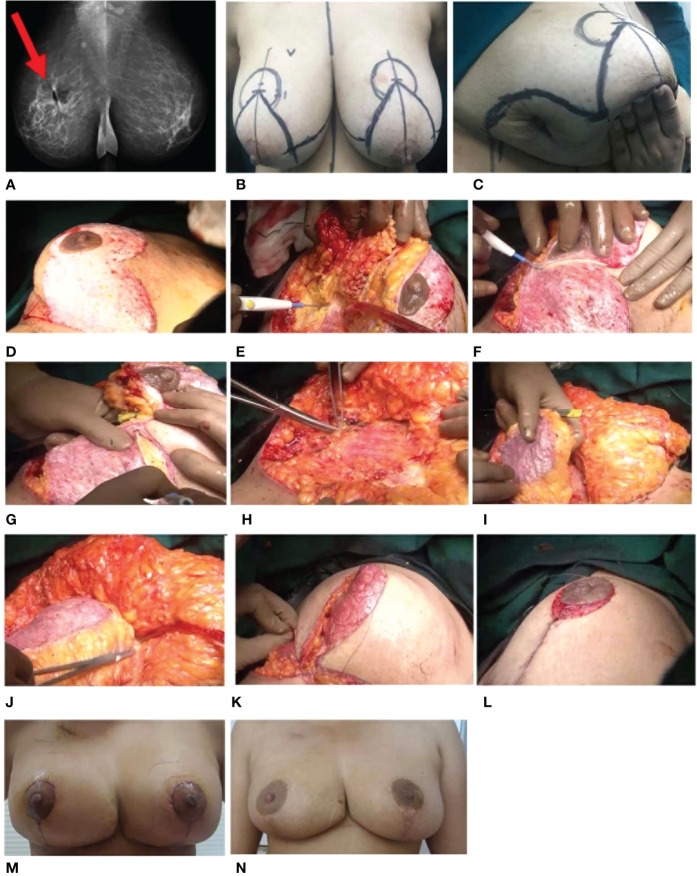
Extreme TM. **(A)** Pre-operative radiology imaging 2D mammogram—MLO view of both breasts. Ill-defined spiculated lesion is seen in the upper deep central quadrant of the right breast, and single enlarged right axillary node is seen. **(B)** Markings. **(C)** Previous lumpectomy scar. **(D–L)** Intra-operative images. **(D)** De-epithelialization of the infero-medial pedicle. **(E)** Excision of the tumor with skin. **(F)** Defining the pedicles. **(G)** Nipple–areolar complex (NAC) is on the superomedial pedicle, and inferior pedicle is defined to be used as a filler. **(H)** Clipping of the tumor bed. **(I)** Inferior pedicle used as a filler in the defect (arrow). **(J)** Inferior pedicle used as a filler and fixed. **(K)** Restructuring of the breast. **(L)** Re-structured breast. **(M, N)**. Post-operative images. **(M)** One-month follow-up. **(N)** Annual follow-up. TM, therapeutic mammoplasty; MLO, mediolateral oblique.

The post-op histopathology report showed unclassified residual IDC with single axillary node positivity. The patient received adjuvant RT followed by an electron boost to the tumor bed. The patient was counseled for adjuvant hormone therapy. The patient tolerated the overall treatment well and is disease-free after 6 years post-oncoplastic surgery (**Figure 7** and **Supplementary Video 3**).

### 6.4 Case IV: Split Reduction Mammoplasty

A patient >50 years with E-cup breasts (Grade III ptosis) presented with a large diffuse lump in the left UOQ measuring 23 × 36 × 34 mm on radiological evaluation. Tru-Cut biopsy suggested IDC Grade II, and IHC revealed ER/PR-positive and HER2-negative status.

The patient was marked for a Wise pattern incision. The inferior pedicle and the medial wing were de-epithelialized, but the lower lateral wing (LOQ) of the IMF incision was omitted. With the use of a separate incision, the whole UOQ was excised with the overlying skin. The dimensions of the specimen were 10 × 8 × 4 cm, 8 × 6 × 2.5 cm, and the weight was 320 cc. The shave margins were sent for frozen-section evaluation to confirm margin negativity. An axillary nodal clearance was performed following a sentinel node biopsy (2/3 nodes) *via* the same incision (2/14 nodes). The superomedial pedicle was dissected, and the de-epithelialized medial part of the lower pedicle was used as a filler. The lateral wing (LOQ) and tumor cavity were connected to create a continuum of the skin. The NAC was positioned on the superomedial pedicle, and the breast tissue was reshaped. After closure, an S-shaped incision was made, termed “split reduction”.

**Figure 8:**
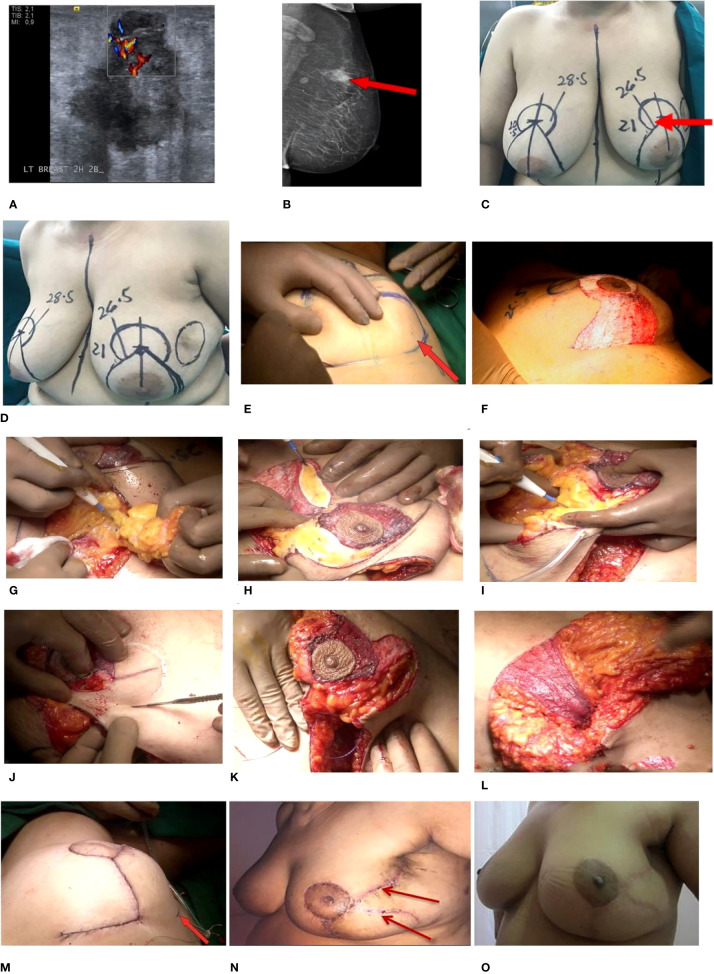
Split reduction TM. **(A, B)** Pre-operative radiology images. **(A)** 2D mammogram—MLO view of the left breast. Oval iso- to hyperdense lesion is seen in the upper quadrant of the left breast, which shows spiculations and a small enlarged left axillary node. **(B)** USG—a hypoechoic solid lesion, taller than wide is seen at the 2 o’clock 2B position of the left breast. **(C, D)** Markings also showing tumor in the UOQ with involved skin (arrow). **(E–M)** Intra-operative images. **(E)** No incision on lower outer part of IMF. **(F)** De-epithelialized inferior pedicle along with the medial wing. **(G)** Excision of the tumor along skin. **(H)** Dual pedicles being defined. **(I)** Delineating the superomedial pedicle. **(J)** Marking of the future NAC. **(K)** Connecting the tumor cavity and the lateral wing to create a continuum of skin. **(L)** Inferior pedicle being used as a filler. **(M)** Restructured breast, with the dermal incision in the lateral aspect taken inadvertently. Post-operative images at **(N)** 1-month follow-up and **(O)** 3-year follow-up. TM, therapeutic mammoplasty; MLO, mediolateral oblique; USG, ultrasonography; UOQ, upper outer quadrant; NAC, nipple–areolar complex.

The post-op histopathology revealed IDC Grade II. The patient received adjuvant RT followed by an electron boost to the tumor bed. The patient’s hormonal therapy was continued. The patient tolerated treatment well and is disease-free after 4 years post-diagnosis. The pre- and post-operative images for this patient are depicted in **Figure 8**, and the technique demonstration is presented in **Supplementary Video 4**.

## Data availability statement

The datasets presented in this article are not readily available because the data collected is of clinicopathological profiles, surgical, oncological, cosmetic, and patient-related outcomes for cases treated at our centre. This data cannot be shared outside the institution unless ethical approval is given by our ethics committee for a specific project. Requests to access the datasets should be directed to CK email: dr.koppiker@prashanticancercare.org.

## Ethics statement

The studies involving human participants were reviewed and approved by PCCM-CTCR Independent Ethics Committee EC/NEW/INST/2021/2443. The patients/participants provided their written informed consent to participate in this study. Written informed consent was obtained from the individual(s) for the publication of any potentially identifiable images or data included in this article.

## Author contributions

CK: This author was involved in the conception and design, financial support, administrative support, manuscript writing, final approval of the manuscript and was generally accountable for all aspects of the work. SJ, RM, DAK: These authors have contributed equally and were involved in the collection and assembly of data, data analysis and interpretation, data visualization, manuscript writing, final approval of manuscript, accountable for all aspects of the work. PC: Data interpretation and manuscript writing. AJ, JJ: Collection of data. SK: Data representation and visualization AB, DT: Manuscript writing. GS, UD, CD, HA, BV: Data generation. SmN: Collection of data. SaN: Editorial help LB: Administrative support. MP: Data interpretation. All authors contributed to the article and approved the submitted version.
